# Virtual Reality and Three-Dimensional Printed Models Improve the Morphological Understanding in Learning Mandibular Sagittal Split Ramus Osteotomy: A Randomized Controlled Study

**DOI:** 10.3389/fsurg.2021.705532

**Published:** 2021-12-22

**Authors:** Henglei Zhang, Yu He, Ying Chen, Jianfeng Liu, Qi Jin, Shixing Xu, Xi Fu, Jia Qiao, Bing Yu, Feng Niu

**Affiliations:** Department of Craniomaxillofacila Surgery, Plastic Surgery Hospital, Chinese Academy of Medical Sciences & Peking Union Medical College, Beijing, China

**Keywords:** sagittal split ramus osteotomy, surgical education, anatomy, virtual reality, three-dimensional printing

## Abstract

**Background:** The mandibular sagittal split ramus osteotomy (SSRO) is a routine operation performed to correct mandibular deformity including mandibular retrusion, protrusion, deficiency, and asymmetry. The SSRO remains a challenging procedure for junior surgeons due to a lack of adequate morphological knowledge necessary for success in clinical practice. Virtual reality (VR) and three-dimensional printed (3DP) models have been widely applied in anatomy education. The present randomized, controlled study was performed to evaluate the effect of traditional educational instruments, VR models, and 3DP models on junior surgeons learning the morphological information required to perform SSRO.

**Methods:** Eighty-one participants were randomly assigned to three learning groups: Control, VR, and 3DP. Objective and subjective tests were used to evaluate the learning effectiveness of each learning instrument. In the objective test, participants were asked to identify 10 anatomical landmarks on normal and deformed models, draw the osteotomy line, and determine the description of SSRO. In the subjective test, participants were asked to provide feedback regarding their subjective feelings about the learning instrument used in their group.

**Results:** The objective test results showed that the VR and 3DP groups achieved better accuracy in drawing the osteotomy line (*p* = 0.027) and determining the description of SSRO (*p* = 0.023) than the Control group. However, there was no significant difference among the three groups regarding the identification of anatomical landmarks. The VR and 3DP groups gave satisfactory subjective feedback about the usefulness in learning, good presentation, and enjoyment. The Control and 3DP groups reported positive feelings about ease of use.

**Conclusion:** The current findings suggest that VR and 3DP models were effective instruments that assisted in the morphological understanding of SSRO-related anatomical structures. Furthermore, 3DP models may be a promising supplementary instrument to bridge the gap between conventional learning and clinical practice.

## Introduction

The mandibular sagittal split ramus osteotomy (SSRO) was first introduced in the 1950s and is considered a milestone in craniomaxillofacial surgery (1). The SSRO enables correction of mandibular deformity in three dimensions, including mandibular retrusion, protrusion, deficiency, or asymmetry (1–3). After decades of technical improvements, the mandibular osteotomy has developed from a life-threatening surgery to a routine operation for craniomaxillofacial surgeons. However, the SSRO remains a challenging procedure for junior surgeons. Complications such as unfavorable splits, fractured segments, temporomandibular joint dysfunction, and inferior alveolar nerve damage prevent the expected successful outcomes. Surgeons must possess adequate anatomical knowledge of the complex irregular anatomy to successfully perform SSRO in clinical practice.

The ideal educational process depends on experienced teachers, a suitable syllabus, optimum facilities, and available teaching instruments (4, 5). Both clinicians and anatomists agree that it is difficult for the traditional mode of anatomical education to meet modern learning requirements (4, 5). Dissection and participation in real surgery is considered the gold standard for students and junior surgeons to learn operation-related anatomy. However, iatrogenic injuries, surgical complications, and ethical dilemmas make it difficult to provide hands-on training in real surgery. Furthermore, there is a gap between traditional textbook learning and real clinical cases. It is difficult for the obscure words and two-dimensional pictures in textbooks to stimulate learning enthusiasm, and for students and junior surgeons to fully understand the surgical procedure using anatomical models with normal structures. To overcome these shortcomings, teacher-centered methods have been replaced by learner-centered and problem-based learning approaches. In this process, the learning instruments are particularly important in improving learning efficacy and stimulating the subjective learning initiative.

The correct understanding of the complex anatomical structures, such as the irregular mandible, is the first step in successfully performing SSRO (6, 7). To achieve this goal, novel models have been widely used in the anatomy teaching field (8–12), including virtual reality (VR), augmented reality (AR), mixed reality (MR), and three-dimensional printed (3DP) models. VR models are designed and fabricated by digital technology to simulate three-dimensional anatomic structures in a virtual world (8). Compared with conventional two-dimensional pictures, the head-mounted VR devices enable users to view stereoscopic images in a fully immersive environment. Several studies have declared that VR models are effective tools to improve the learning of complex spatial structures because of visual advantages with fast processing, relative reliability, and parallelism (8, 11, 13–17). However, the interactive VR experience lacks the tactile information that is crucial to effectively master the complex structures and perform the surgical operation. The 3DP technology creates vivid life-sized models with complex spatial structures from digital data (10). The tangible 3DP models may improve learning efficacy by providing tactile feedback. VR and 3DP models have been used in many clinical specialties for advanced teaching, preoperative planning, and simulated surgery (14, 18–23). However, the effectiveness of VR and 3DP models in learning to perform SSRO remains unclear. Therefore, the aim of the present study was to evaluate the effect of VR and 3DP models on the attainment of an operation-related morphological understanding of SSRO for junior surgeons.

## Materials and Methods

### Study Design and Participants

The present study was approved by the Institutional Review Board of our hospital. The prospective, randomized, controlled trial was carried out at our institute. First-year plastic specialists who had completed a 3-year standardized surgical resident training program were recruited to participate from September 2020 to February 2021. The participants at this training stage have a fundamental understanding of surgical anatomy structures; however, they have only basic knowledge of craniomaxillofacial surgery, without a comprehensive understanding of and sufficient experience with SSRO. The participants had a similar medical background and participated voluntarily; their decision to participate and their grade in the present study did not influence their performance evaluation. Eighty-one participants (36 women and 45 men) were recruited. The participants were randomly divided into three learning groups: Control (*n* = 27, 12 women and 15 men, mean age 27.9 years old), VR (*n* = 27, 12 women and 15 men, mean age 27.5 years old), and 3DP (*n* = 27, 12 women and 15 men, mean age 28.1 years old).

### Learning Strategy

**Figure 1:**
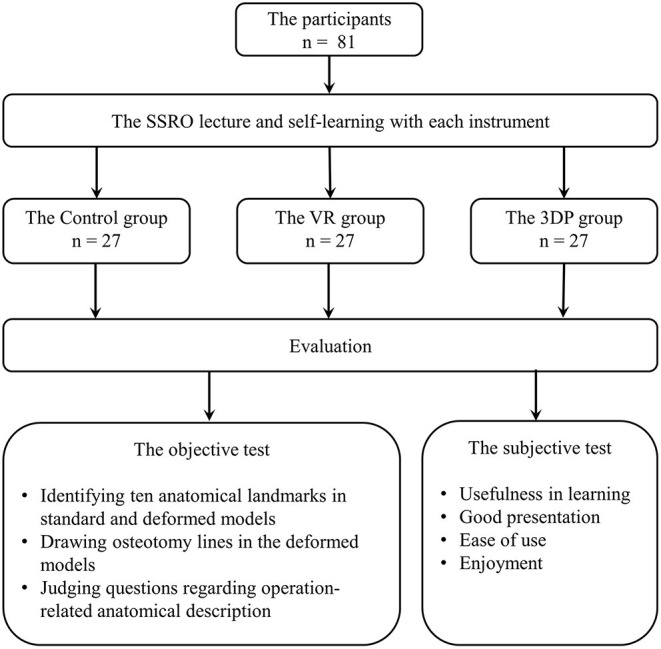
The brief overview of the learning strategy in the three groups.

Figure 1 shows a brief overview of the learning strategy in the three groups. The three learning groups studied mandibular SSRO separately. All participants first attended a lecture on the fundamental knowledge required for SSRO. Professors with ample educational experience described the craniomaxillofacial anatomical landmarks, operation-related anatomical structures, and their spatial relationships. The participants were then asked to review the content related to SSRO using textbooks, imaging data, and the learning instrument specific to their group. There was no difference in the learning time among three groups.

**Figure 2:**
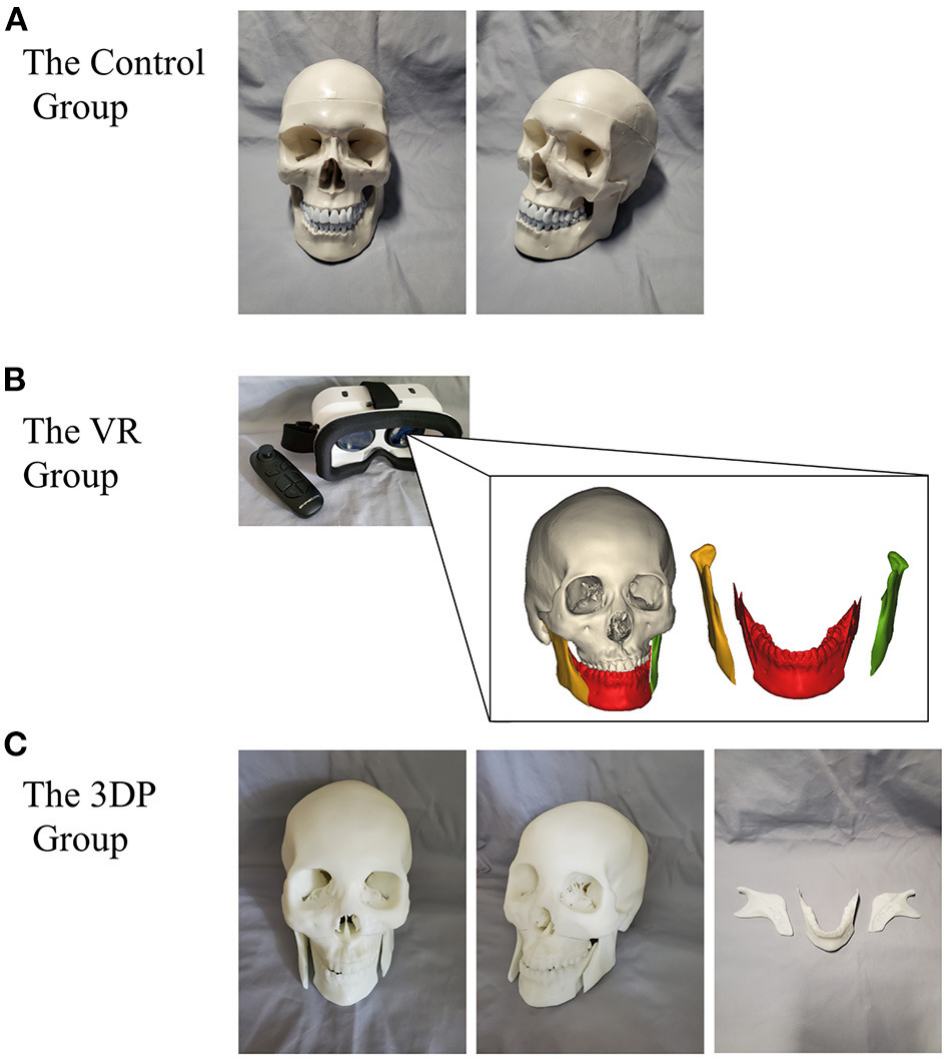
The concise exhibition of the learning instruments used in the Control group **(A)**, VR group **(B)**, and 3DP group **(C)**.

**Figure 3:**
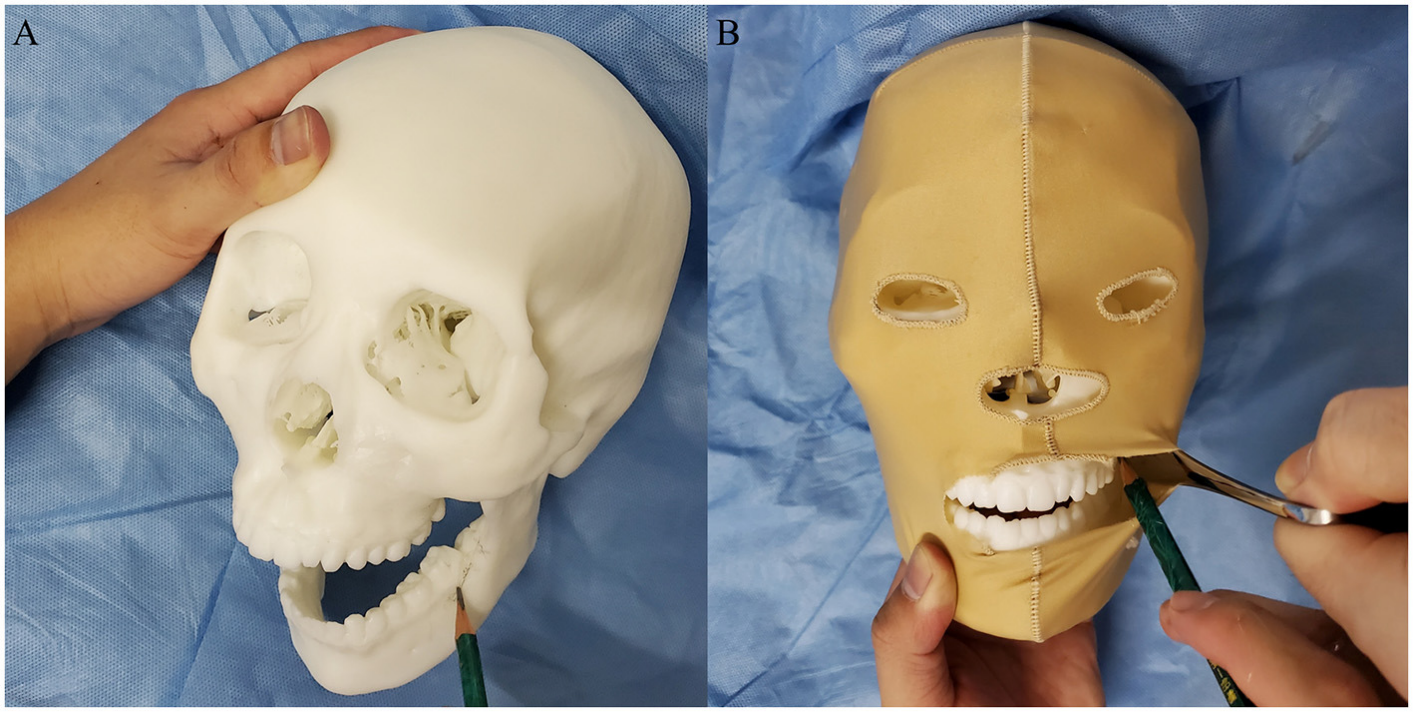
The participates in 3DP group could observe, touch, and draw osteotomy lines on the models to fully comprehend the spatial structures **(A)**. The models were wrapped in elastic headgear to simulate soft tissues **(B)**. The participants could lift the elastic headgear to roughly observe the surgical exposure field.

Figure 2 concisely displays the learning instruments used in the three groups. The Control group experienced the traditional learning method using textbooks and standard anatomy models to learn operation-related anatomical information; participants drew the osteotomy lines on a standard model of the intact normal craniomaxillofacial structures. Based on the same information used to teach the Control group, the VR group used head-mounted devices to experience virtual models to assist in learning the relevant anatomy. CT data from real clinical cases were applied to reconstruct the VR models. The immersive experience with virtual non-deformed and deformed craniomaxillofacial bones was provided by a VR system (VR Shinecon, Shinecon Co., Ltd., China). In the learning process, the VR group used a control device to move, resize, and rotate the VR models to observe the key structures. The 3DP group used life-sized models of real deformed cases based on the traditional method to improve their morphological understanding. CT reconstruction data were inputted into a 3DP system platform (SLA660, Aidi Co., Ltd., China) to build the 3DP models. The 3DP group could observe, touch, and draw osteotomy lines on the models to fully comprehend the spatial structures (Figure 3). The models used in Control group and 3DP group were wrapped in elastic headgear to simulate soft tissues. The participants could lift the elastic headgear to roughly observe the surgical exposure field.

### Evaluation

An objective test of operation-related anatomical knowledge and a subjective questionnaire regarding the learning experience were performed to assess the effectiveness of the different learning instruments. The cases with models and imaging data used in the evaluation differed from the cases used in the learning process.

The objective evaluation included three parts. In the first part, the participants were asked to identify the following 10 anatomical landmarks on standard models with normal structures and on deformed models with mandibular asymmetry: the mandibular condyle, the coronoid process of the mandible, the posterior border of the mandibular ramus, the mandibular lingula, the mandibular foramen, the sulcus of the mandibular nerve, the surface projection of the mandibular nerve canal, the mental foramen, the external oblique line of mandible, and the mandibular first molar. In the second part, the participants were asked to draw osteotomy lines on a deformed model of mandibular retrusion. In the third part, the participants were asked to judge whether the following descriptions of operation-related anatomical description were correct. The questions were selected from the examination database of craniomaxillofacial surgery and determined by three experienced professors together (Prof. Feng Niu, Prof. Ying Chen, and Prof. Lai Gui). Q1: The surgical incision is made from lingual to the external oblique line, halfway up the mandibular ramus superiorly to mesial of the first/second molar inferiorly; Q2: The medial osteotomy line is angled parallel to the inferior border of the mandible and terminated posterior to the lingula into the fossa; Q3: The buccal osteotomy should start at the lower border and include the lingual cortex. If the lingual cortex of the lower border cannot be included in the osteotomy, an unexpected large split will occur; Q4: The presented fracture lines of buccal plate fracture, buccal plate fracture including the coronoid process, fracture that does not reach the lingula, and retromolar fracture are considered bad splits; and Q5: In the process of splitting the mandible, surgeons should ensure that the intact neurovascular bundle is not attached to the distal segment. Furthermore, the participants were asked to modify the description that they thought was the most incorrect. The accuracy of the answer in each part was recorded and analyzed.

The subjective learning experience was investigated to understand the participants' attitudes to their learning instruments. The questionnaire included four aspects: usefulness in learning, good presentation, ease of use, and enjoyment. The 5-point Likert scale (strongly agree, agree, indifferent, disagree, and strongly disagree) was used to assess the participants' subjective feelings.

### Statistical Methods

The statistical analyses were performed by SPSS 19.0 software (IBM Corp, Armonk, New York). The Kolmogorov-Smirnov test was used to determine the normality of distribution of the variables. The average values among three groups were compared by analysis of variance testing. The statistical significance of qualitative data was determined by the chi-squared test. The Mann-Whitney *U* test was applied to evaluate the subjective results. The significance level was set at *p* < 0.05.

## Results

### Objective Test Results

**Figure 4:**
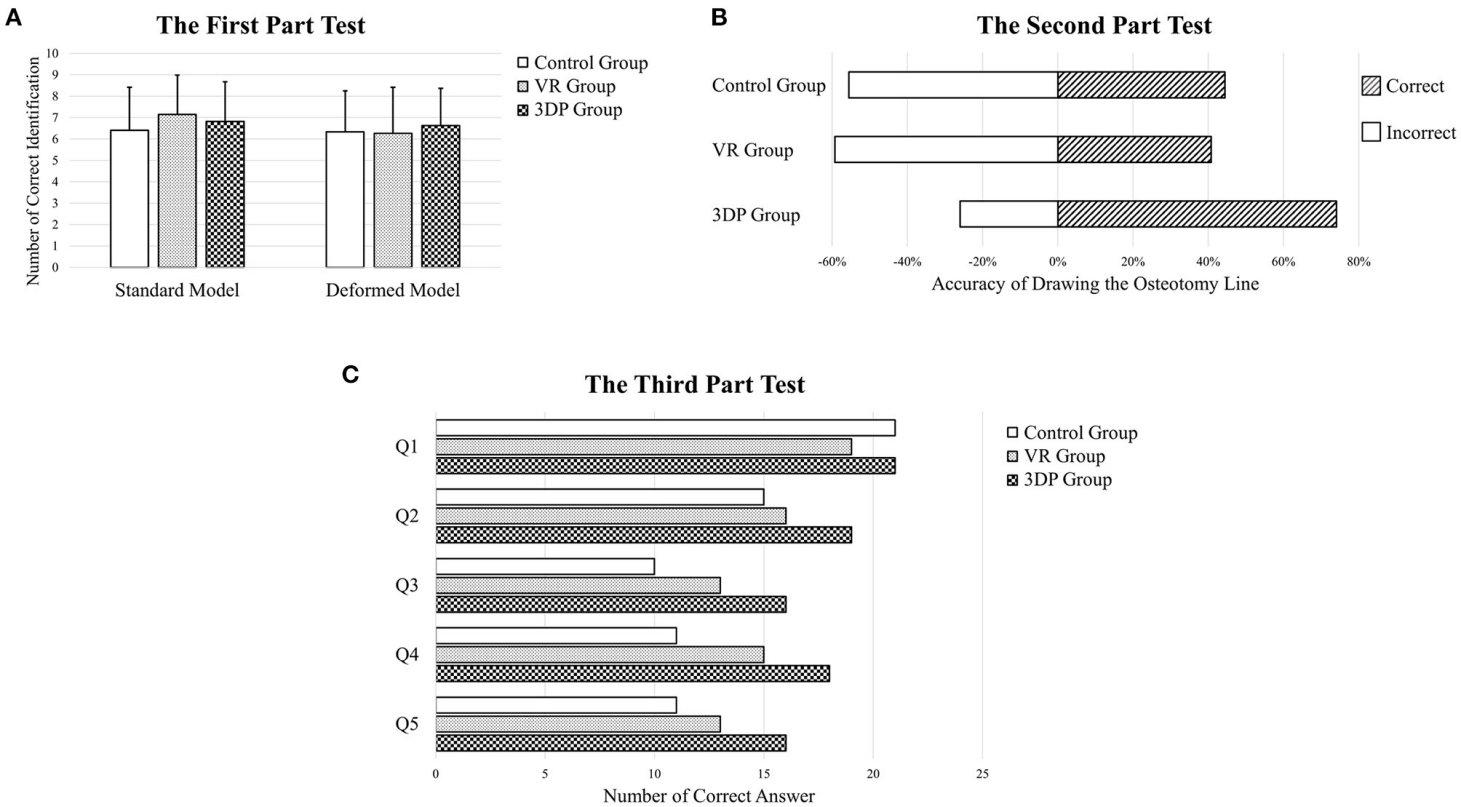
The first part **(A)**, second part **(B)**, and third part **(C)** objective test results about learning mandibular sagittal split ramus osteotomy.

The objective test results are displayed in Figure 4. The 3DP and VR groups generally showed better results in the objective tests than the Control group.

The results of the first part of the objective test showed that there was no significant difference among the three groups regarding the identification of 10 anatomical landmarks on the non-deformed and deformed models. The number of landmarks correctly identified on the non-deformed models was 6.407 ± 2.005, 7.148 ± 1.916, and 6.815 ± 1.8613 in the Control, VR, and 3DP groups, respectively; the number of landmarks correctly identified on the deformed models was 6.333 ± 1.840, 6.259 ± 2.159, and 6.630 ± 1.735, respectively. The anatomical landmarks with the lowest identification accuracy were the mandibular lingula, the sulcus of the mandibular nerve, and the surface projection of the mandibular nerve canal.

The 3DP group achieved the best results in the second part of the objective test. The osteotomy lines were drawn accurately by 20 participants in the 3DP group, compared with 12 and 11 participants in the Control and VR groups (*p* = 0.027).

In the third part of the objective test, the 3DP group answered more questions correctly (66.667%) than the Control and VR groups (50.370% and 56.296%, *p* = 0.023). The overall percentages of correct answers for each the five questions were 75.309, 61.728, 48.148, 54.321, and 49.383%, respectively.

### Subjective Test Results

**Figure 5:**
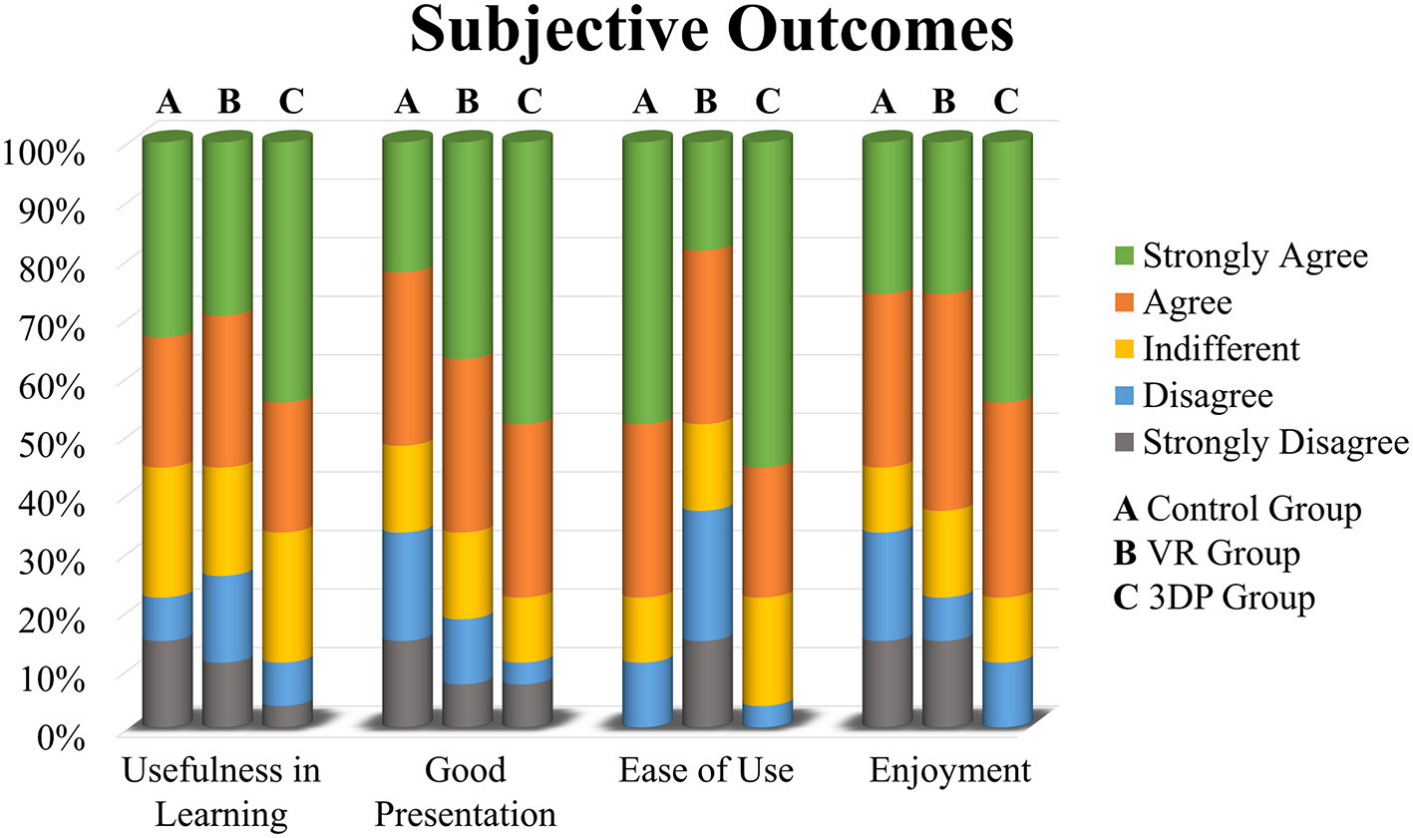
The subjective feedback regarding the learning instruments used to obtain a morphological understanding of sagittal split ramus osteotomy in the Control group **(A)**, VR group **(B)**, and 3DP group **(C)**.

The subjective feedback regarding the learning instruments used to obtain a morphological understanding of SSRO is presented in Figure 5. Regarding usefulness in learning, the 3DP group gave a high appraisement, with 66.667% responding “Agree” or “Strongly Agree.” The 3DP and VR models achieved a good presentation of the anatomical morphology of SSRO. Six, 10 and 13 participants in the Control, VR, and 3DP groups, respectively, strongly agreed that their instruments were well able to present SSRO-related knowledge. Many participants in the VR group believed that the VR devices lacked ease of use, with 37.037% responding “Disagree” or “Strongly Disagree”; in contrast, only three and one participants chose “Disagree” in the Control and 3DP groups. The 3DP group believed that the learning instruments were enjoyable, with no participants choosing “Strongly Disagree.” Overall, the 3DP and VR groups gave positive subjective feedback regarding their learning instruments.

## Discussion

Medical education has progressively changed in recent decades. The application of traditional education methods, such as cadaver surgery, prosections, and theater-based learning, is limited by many factors (4, 5). Novel educational instruments are constantly emerging and will gradually make up for the shortcomings of traditional methods. The present study demonstrated that VR and 3DP models were effective supplementary instruments in learning the SSRO-related morphology. Moreover, 3DP models provided interactive tactile feedback and stimulated subjective learning initiative, which helped junior surgeons better understand the spatial relationships between anatomic structures.

Digital models are novel instruments that have been applied in the educational field. Several studies have declared that VR models are an effective tool for learning anatomy and simulating surgery (8, 11, 14, 15, 17). Izard et al. reported that the application of operational VR models of the cranium and cranial fossa helps with medical training and improves surgical skills (8). Pfaff et al. believed that VR surgical planning in standardized cases, such as SSRO, may be a useful ancillary resource for training junior surgeons (13). Moreover, VR plays an important role in surgical training and simulation as a spatial vision resource. VR instruments have become the primary training method for junior surgeons in the fields of plastic surgery, orthopedics, thoracic surgery, and neurosurgery. However, one obvious drawback of VR devices is the lack of tactile feedback that improves the understanding of spatial relationships. Some studies have integrated VR and tactile feedback devices to enhance the learning effect (24, 25). Theoretically, such integration with multisensory stimulation promotes spatial comprehension. However, integrated devices are currently unavailable in our institute. In the present study, the VR group generally achieved better objective test results than the Control group. However, the VR devices did not show many advantages in the first part of the test in which the questions were relatively simple. Further study is required to determine whether novel educational tools are necessary in learning simple anatomical knowledge. In general, VR models could be used to supplement traditional learning to improve the understanding of complex anatomy.

In the learning process, the trainees build anatomical structure representations that rely on the visual and tactile senses to attain an understanding of the spatial locations. Vision plays the most important role in learning anatomic morphology because of its fast processing, relative reliability, and parallelism. However, it is inadequate to only use the visual sense to learn complex structures and surgical procedures. The 3DP models meet the participants' demand for multisensory stimulation. Studies have found that 3DP models with tactile sensation play an active role in the process of learning complex structures, such as acetabular fractures, liver structures, and craniovertebral junction deformities (19, 21, 26). Licci et al. introduced a synthetic 3DP simulator which allowed trainees to develop skills for endoscopic ventricular lesion removal (23). The authors evaluated the validity of the 3DP model in term of realism, mechanical proprieties, procedural content, and handling. They found that the simulator was a useful training instrument to teach neuroendoscopic techniques and support the development of the required surgical skills. These studies not only focused on the traditional method of anatomic learning, but also on the disease-related structures. In the current study, we found that the 3DP models were helpful in enabling junior surgeons to understand the descriptions contained in textbooks. In the third part of the test, the 3DP group showed excellent results that were superior to the other groups, suggesting that 3DP models may be a promising method with which to improve the understanding of complex structures as a link between textbook information and clinical cases. The physical communication between participates and 3DP models is considered to be the potential reason for satisfactory results. In the future, the multi-sensory stimulation learning will be the mainstream in medical education. Tactile is the complex information obtained in the physical interaction between skin and external environment. Surgeons can directly touch the patients' anatomical structures through their own hands to obtain a great wealth of tactile information, such as morphology, spatial relationship, texture and so on. The physical interaction obtained by touch is more conducive to trainees for understanding anatomical structures. The 3DP models can be used to bridge the gap between anatomical knowledge and clinical practice requirements. Combined with textbooks and multiple models, the learning process has experienced a transition from physiomorphology to pathomorphology, from plane to stereo, and from vision to multisensory. In theory, actively touching anatomical landmarks and the osteotomy region promotes the learning of SSRO.

The subjective initiative of junior surgeons and students is necessary in medical education due to the highly specific training and profession. Active and engaging learning instruments and strategies may be beneficial in increasing trainees' level of learning interest. In the present study, the VR and 3DP models motivated junior surgeons to become deeply involved in the learning process. The VR and 3DP groups gave satisfactory subjective feedback about usefulness in learning, good presentation, and enjoyment. However, the subjective feedback showed that the VR devices were not considered easy to use. It cannot be ruled out that the unsatisfactory outcomes of VR group were caused by insufficient equipment usability rather than visualization modality. Considering this limitation, the application experience of VR devices should be improved before educational application. Overall, the reason that the novel models promoted subjective initiative in SSRO learning may be that they were considered interesting, useful, and provided a link between theory and practice.

In this era of technological prosperity, the surgical training equipment is constantly changing. The VR, AR, MR technology have made great development in recent years, which offer opportunities to promote medical education and improve patients' prognosis. AR technology can be described as a view of the real-world environment which is enhanced by digital image, video, audio, even touch and smell. MR technology can integrate the physical world and digital world to create a new environment for real-time interaction between physical and digital objects. The advanced AR and MR devices provide a digital environment that realistically simulates the actual pathological morphology and surgical environment for specific diseases. With the research and application of multi-sensory technology, the advanced digital technology will have a positive impact on medical education and clinical practice to promote quality of care and improve patients' outcomes in the future.

The present study had some limitations. First, the long-term knowledge retention of SSRO-related anatomic morphology was not evaluated. The purpose of the current study was to understand the effectiveness of novel models in promoting learning. In theory, positive understanding is helpful for long-term knowledge retention. Second, the small sample size and non-compulsory recruitment with low motivation may have led to research bias. The medical background of participates could not be exactly the same, even though we had a preliminary screening. However, the randomized controlled design was applied to minimize bias. Third, the questionnaires used in current study was designed by three experienced professors of craniomaxillofacial surgery. However, the questionnaires without pre-experimental data have not been strictly evaluated, which may lead to research bias. Fourth, the models of real deformed cases did not include important soft tissues, such as blood vessels and nerves, which affect the surgical outcomes. The reconstruction of arteries and nerves in real deformed cases requires examination of CT angiography and nuclear magnetic resonance, which are not part of routine preoperative examination. The soft tissue anatomy was emphasized in the lectures to make up for these shortcomings. Fifth, headset VR equipment with single visual stimulation was used in the present study. Advanced VR devices with multisensory intervention may provide better learning results; however, such equipment is not currently available in our institute.

In conclusion, the present results demonstrated that 3DP models were effective in assisting junior surgeons to obtain a morphological understanding of SSRO-related anatomical structures. The VR models also achieved good outcomes in anatomical structures learning. We believe that the novel model is a promising supplementary instrument to bridge the gap between conventional learning and clinical practice.

## Data Availability Statement

The raw data supporting the conclusions of this article will be made available by the authors, without undue reservation.

## Author Contributions

FN and YH conceived the study, designed the study protocol. YH and HZ analysed the data and wrote the initial draft of the manuscript. YC, SX, JQ, XF, QJ, JL, and BY were involved in implementing trial and data collection. FN and HZ edited the final draft of the manuscript. All authors contributed to the article and approved the submitted version.

## Funding

This work was supported by the Clinical and Translational Medicine Research Project, Science and Technology Innovation Project of Medicine and Health, Chinese Academy of Medical Sciences (2020-I2M-CT-B-078) and the Beijing Natural Science Foundation Program (No. 7192180).

## Conflict of Interest

The authors declare that the research was conducted in the absence of any commercial or financial relationships that could be construed as a potential conflict of interest.

## Publisher's Note

All claims expressed in this article are solely those of the authors and do not necessarily represent those of their affiliated organizations, or those of the publisher, the editors and the reviewers. Any product that may be evaluated in this article, or claim that may be made by its manufacturer, is not guaranteed or endorsed by the publisher.
